# The Quinine Curse

**Published:** 1888-02

**Authors:** Wm. Jefferson Guernsey


### THE QUININE CURSE.

Did any member of the Hahnemannian Association ever notice how much more finger-soiled the corks of some of his vials become than others? Think over your list of medicines before looking and you will, in all probability, be ready to declare that you have not more frequently used, or shown partiality for, any particular drug, yet pull out the drawer, and lo! the evidence is before you. Now open a repertory and turn to the symptom "aggravation from Peruvian bark," and I wager you will find every name of the soiled-cork brigade recorded therein.

To be more explicit, I do not think that I know any more about *Pulsatilla* than any other remedy, and I am sure that I steer clear (with conscientious dread) of any pet prescription of that or any other medicine for such and such ailments, yet I have noticed that I give *Puls.* oftener than any other medicine. Why is this, and why are the anti-quinine medicines so frequently employed? I believe it is from the fact that that cursed drug is so commonly used by all our patients, on all occasions, for all complaints. In short, it is the fashionable thing now to take it to prevent a cold, to cure a cold, and to keep you cold (by breaking a fever).

I believe we should study closely the list of medicines that antidote this drug, and perhaps thus find curative agents for multitudes of stubborn cases.

I need hardly add, that though the medicines may have been given without antidotal intention thus far that the beauty of the infallible law requiring their exhibition is noteworthy.

WM. JEFFERSON GUERNSEY, M. D.

January 10th, 1888.

